# Carboxymethylcellulose excipient allergy: a case report

**DOI:** 10.1186/s13256-021-03180-y

**Published:** 2021-11-24

**Authors:** Katherine Townsend, James Laffan, Grant Hayman

**Affiliations:** Immunology and Allergy, St Helier University Hospital NHS Trust, D Block 2nd Floor, Wrythe Lane, Carshalton, SM5 1AA UK

**Keywords:** Allergy, Hypersensitivity, Excipient, Carboxymethylcellulose (CMC), Case report

## Abstract

**Background:**

Excipients are widely used in pharmaceuticals, detergents, food, and drink because of their properties of low toxicity and hypoallergenicity. The excipient carboxymethylcellulose is used extensively as a thickener in foods such as baked goods, ice cream, gluten free, and reduced fat products, where it may be labeled as e-number E466. However, excipients can rarely cause type 1 hypersensitivity reactions. Several publications have described systemic allergy following carboxymethylcellulose exposure in pharmaceuticals, particularly systemic corticosteroids. Furthermore, there is one reported case in the literature of anaphylaxis following food containing carboxymethylcellulose.

**Case presentation:**

We identify a case of anaphylaxis in a 45-year-old atopic Caucasian woman on receiving an injectable suspension of the corticosteroid triamcinolone acetonide containing carboxymethylcellulose, and subsequent allergic symptoms on reexposure to carboxymethylcellulose in a commercial drink. Diagnosis of carboxymethylcellulose excipient allergy was confirmed through skin prick testing using Celluvisc carmellose 0.5% eye drops, which contain carboxymethylcellulose as the active ingredient.

**Conclusion:**

This case highlights the importance of identifying excipients such as carboxymethylcellulose as causes of allergy, to reduce burden of further hypersensitivity reactions, not just to drugs but to other consumables.

## Introduction

Excipients are inactive substances formulated alongside active ingredients, often serving as vehicles for a drug [1]. However, they may act as hidden allergens, and are known to be causal in drug type I hypersensitivity reactions. Excipient allergy may be difficult to diagnose. The excipients carboxymethylcellulose and polyethylene glycol have previously been shown to be significant causes of type I hypersensitivity reactions in a sample of patients with a confirmed diagnosis of allergy to systemic corticosteroid preparations [2].

Carboxymethylcellulose is a derivative of the plant polysaccharide cellulose. It is synthesized through an alkali-catalyzed reaction in which carboxymethyl side groups are substituted on to the cellulose backbone, rendering it soluble. It is widely used for its high viscosity and solubility along with its expected low toxicity and hypoallergenicity, given its derivation from inert plant material [3]. Applications include as a thickener or viscosity modifier, binder, stabilizer, lubricant, or gelling agent [4]. It is present in multiple pharmaceuticals, paints, detergents, foods, and drinks. In foods it may be labeled as e-number E466, or E469 when enzymatically hydrolyzed. It is used extensively in gluten free and reduced fat products, as well as in baked goods, spreads, and ice creams. Several case reports of systemic allergy have been described following its exposure in pharmaceuticals, including systemic corticosteroid preparations, [2, 5–9] barium enema, [10] and carmellose sodium [11]. There is also one reported case of anaphylaxis in a child following food containing carboxymethylcellulose [12].

In this case, we identify excipient allergy in a patient who developed systemic anaphylaxis on receiving Kenalog, an injectable suspension of the corticosteroid triamcinolone acetonide, and who subsequently manifested allergic symptoms on re-exposure to the excipient in a commercial drink.

## Case presentation

A 45-year-old atopic Caucasian woman developed widespread urticaria and presyncope 30 minutes after receiving an intraarticular elbow injection of Kenalog and lidocaine local anesthetic. She called an ambulance and presented to the emergency department, where physical examination demonstrated generalized urticaria, angioedema of her tongue and throat, and diaphoresis. She was hypotensive and tachycardic; her systolic blood pressure was 50 mmHg and pulse rate was 110. Examination and observations were otherwise normal, with Glasgow Coma scale (GCS) of 15/15. No further investigations were performed. She was treated with 500 μg of 1/1000 intramuscular adrenaline, 10 mg of intravenous chlorphenamine, 200 mg of intravenous hydrocortisone, and 1 L of intravenous 0.9% saline salt, to good effect. She was monitored overnight, received further intravenous hydrocortisone the following morning and was discharged with a course of oral prednisolone 30 mg daily and chlorphenamine 4 mg three times daily for 5 days, to which she clinically responded well.

She had no previous history of similar reactions. She had never had a steroid injection prior to this episode. She had previously used over the counter eye drops for dry eyes, with no history of allergic symptoms. Her past medical history otherwise comprised tennis elbow, mild seasonal allergic rhinitis, blepharitis, and childhood asthma. She was on no regular medications. She had no relevant family history. Regarding social history, she was an ex-smoker with a 5 pack year history, drank minimal alcohol, and worked as a school secretary. She lived with her husband, one son, pet cat, and stables with several horses.

On review in the allergy clinic, skin prick testing was positive to Adcortyl but negative to lidocaine and dexamethasone. Adcortyl and Kenalog are chemically similar injectable suspensions, containing the same active agent and excipients: triamcinolone acetonide; benzyl alcohol, polysorbate 80, sodium carboxymethylcellulose, sodium chloride, and water. At that time, on the basis of available evidence, she was felt most likely to have reacted to the excipient carboxymethylcellulose. She was advised to avoid both triamcinolone acetonide and any substances containing carboxymethylcellulose, provided with an anaphylaxis management plan, and trained with and prescribed two adrenaline autoinjectors.

Three years later, she developed widespread urticaria, chest tightness, light-headedness, and swelling of the lips and tongue within 40 minutes of drinking a supermarket-bought white hot chocolate powdered drink. She again presented to the emergency department, and examination again demonstrated whole body urticaria and facial angioedema, with otherwise normal presentation. Observations revealed mild tachycardia only; she was normotensive on this occasion. No further investigations were performed. She was managed with 10 mg of intravenous chlorphenamine and 200 mg of intravenous hydrocortisone, with good response. She was not given intramuscular adrenaline. She was monitored in the department for several hours and discharged with a 2 day course of prednisolone 40 mg. The white hot chocolate was found to contain e-number E466, an alternative descriptor for carboxymethylcellulose. Skin prick testing was positive to hot chocolate powders containing E466/carboxymethylcellulose, and to Celluvisc carmellose 0.5% eye drops, which contains cross-linked carboxymethylcellulose as its active ingredient.

**Fig. 1:**
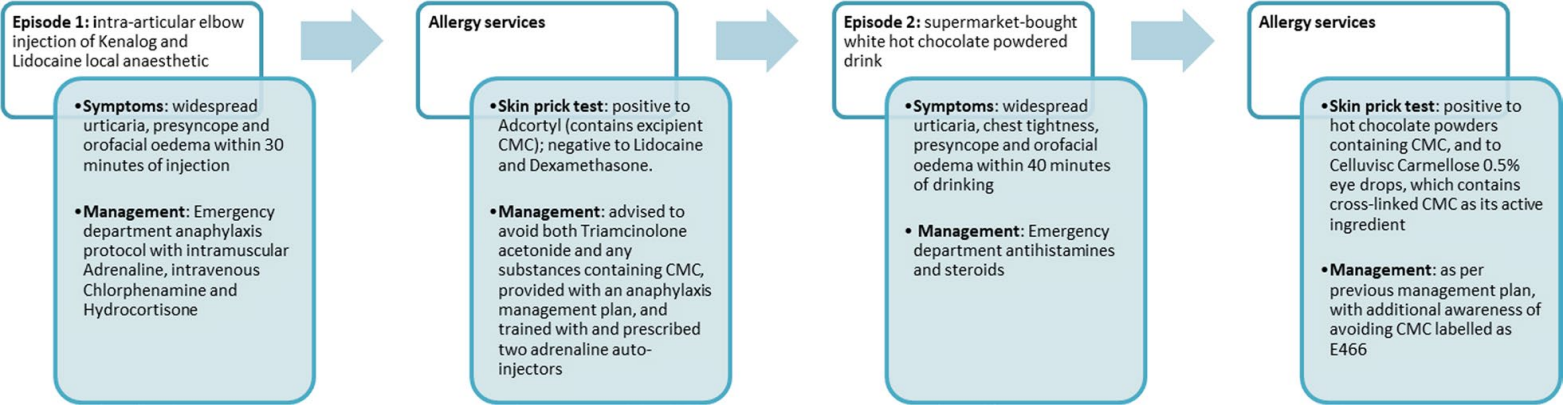
Illustration of patient case history timeline

Her diagnosis was confirmed to be carboxymethylcellulose allergy. Since the above reaction, she has successfully avoided carboxymethylcellulose/E466 in all food, drink, and medications, with no further allergic reactions. On follow-up in allergy clinic, she has developed features of oral allergy syndrome with localized oral itching on eating raw apple, nectarines, peaches, pears, and cherries. Skin prick tests confirmed sensitization to mixed tree pollen, birch tree pollen, mixed weed pollen, and fresh apple on prick-to-prick testing. She has otherwise remained well, with no further food-induced systemic reactions for the subsequent 2 years (Fig. 1).

## Discussion and conclusions

This case highlights excipient carboxymethylcellulose as a potential hidden allergen, not just in medications but in other consumables. It is unique in identifying allergy to a food containing the excipient after demonstrated sensitization to the excipient in a drug. Furthermore, it calls attention to the varying nomenclature of excipients such as carboxymethylcellulose (CMC), also known as E466, or E469 when enzymatically hydrolyzed, which can present difficulties for affected patients trying to avoid further allergen exposure.

Anaphylaxis to excipients are uncommon. There are few case reports identifying carboxymethylcellulose as causative in allergic reactions to systemic corticosteroid preparations, as in our patient [2, 5–9] Only one other case report describes allergy following consumption of food and drink containing carboxymethylcellulose: an ice lolly and a commercial half-frozen beverage (Cafe au lait Swirkle) [12]. Interestingly, in this case, allergic symptoms were delayed by three to four hours, including on directly observed oral challenge with the ice lolly. The authors discuss that as humans lack the enzyme cellulase to digest carboxymethylcellulose, metabolites are only absorbable in small quantities dependent on gastro-intestinal microbial digestion. Notably, their patient had eaten large meals along with the ice lolly, which may also have contributed to a delay in gut absorption and thus allergic reactions. They also highlight increased quantity of this excipient in the drink compared to the ice lolly, which correlated with increased severity of reaction. In our patient, she had not eaten anything else at the time, and her reaction occurred within 40 minutes.

Systemic allergy is potentially life-threatening, presenting a significant burden on both physical and mental well-being of the affected individual. In this case, the identification of CMC/E466 as the cause was a “huge relief,” allowing the return to a sense of normality in daily life.

This case highlights the excipient carboxymethylcellulose as a potential hidden allergen. Importantly, cellulose-containing eye drops provide a readily available and practical way to investigate for allergy in skin prick testing [13]. It may be reasonable to consider this as part of a “hidden allergen panel” in patients with similar histories of food reactions or “idiopathic anaphylaxis.” The case also demonstrates the importance of identifying excipients such as carboxymethylcellulose as causes of allergy, to reduce burden of further hypersensitivity reactions, not just to drugs but to other consumables.

## Data Availability

Not applicable.

## Abbreviations

CMC: Carboxymethylcellulose
